# Supplementary material for: Genomic Analysis of Carbapenem-Resistant Acinetobacter baumannii Isolated from Bloodstream Infections in South Korea
Source: Antibiotics (Basel). 2024 Nov 23;13(12):1124. doi: 10.3390/antibiotics13121124 (PMC11672490; doi:10.3390/antibiotics13121124)
Supplement: Supplementary file 1 [file antibiotics-13-01124-s001.zip › antibiotics-3298187-supplementary.pdf]

Table S1. Genetic characteristics of 12 carbapenem-resistant *A. baumannii* isolates from bloodstream infections.

Strain No.	ST	Contig size	N50	Total coverage /Illumina	Total coverage /Oxford	GC content(%)	Predicted protein-coding sequence (CDS)	tRNA	rRNA
B20AB10	191	4093102	3994026	571.239	117.419	38.94	3883	73	18
A20AB01	195	4109473	4027555	542.311	123.637	38.93	3903	73	18
B20AB06	357	3856432	3856432	676.713	197.094	39.02	3635	74	18
E20AB39	369	4130311	4023022	689.427	110.655	38.95	3945	73	18
C20AB01	451	3958574	3958574	588.923	220.481	39.03	3721	73	18
E20AB21	469	4030517	3948599	524.207	150.766	38.95	3825	73	18
C20AB05	491	3971271	3954338	707.341	121.715	39.11	3734	73	18
D20AB03	784	4025288	3986493	672.717	345.695	39.1	3849	73	18
D20AB01	862	4205227	4121232	677.051	162.452	38.86	3958	73	18
A20AB02	1933	3963066	3935091	601.092	145.984	38.99	3757	73	18
G20AB22	2929	3960514	3868411	661.731	122.739	39.1	3727	73	18
G20AB08	3326	3948245	3948245	535.793	254.994	39.06	3697	72	18

Table S2. MLST analysis of 12 carbapenem-resistant *A. baumannii* isolates from bloodstream infections.

Strain No.	Oxford MLST	*gltA*	*gyrB*	*gdhB*	*recA*	*Cpn60*	*gpi*	*rpoD*	GenBank Accession No.
B20AB10	ST191	1	3	3	2	2	94	3	CP142895-CP142898
A20AB01	ST195	1	3	3	2	2	96	3	CP143268-CP143270
B20AB06	ST357	1	12	3	2	2	145	3	CP142101
E20AB39	ST369	1	3	3	2	2	106	3	CP142797-CP142802
C20AB01	ST451	1	3	3	2	2	142	3	CP142102
E20AB21	ST469	1	12	3	2	2	103	3	CP142658-CP142660
C20AB05	ST491	10	53	4	11	4	98	5	CP143262-CP143263
D20AB03	ST784	1	3	3	2	2	107	3	CP142645-CP142648
D20AB01	ST862	31	33	67	40	1	142	7	CP142642-CP142644
A20AB02	ST1933	1	62	3	2	2	202	3	CP145430-CP145436
G20AB22	ST2929	10	3	4	11	4	98	5	CP146779-CP146826
G20AB08	ST3326	1	102	3	2	2	193	3	CP146231

Table S3. Antimicrobial susceptibility profiles of 12 carbapenem-resistant *A. baumannii* isolates from bloodstream infections.

Strain No.	ST	Amikacin		Gentamicin		Tobramycin		Minocycline		*Tigecycline		Imipenem		Meropenem		*Colistin	
		DISK	**RIS	DISK	RIS	DISK	RIS	DISK	**RIS	DISK	RIS	MIC	RIS	MIC	RIS	MIC	RIS
B20AB10	191	6	R	6	R	6	R	27	S	20	S	32<	R	32<	R	2	S
A20AB01	195	6	R	6	R	6	R	19	**S**	19	S	32<	R	32<	R	2	S
B20AB06	357	19	S	16	S	18	S	21	**S**	20	S	32<	R	32<	R	2	S
E20AB39	369	6	R	6	R	6	R	21	S	17	R	32<	R	32<	R	128<	R
C20AB01	451	6	R	6	R	6	R	21	**S**	19	S	32<	R	32<	R	2	S
E20AB21	469	6	R	6	R	6	R	28	S	20	S	32<	R	32<	R	2	S
C20AB05	491	9	R	6	R	18	S	26	S	20	S	32	R	32<	R	2	S
D20AB03	784	6	R	6	R	6	R	25	S	20	S	32<	R	32<	R	2	S
D20AB01	862	16	I	13	I	15	S	30	S	20	S	32<	R	32<	R	2	S
A20AB02	1933	20	S	19	S	18	S	23	**S**	20	S	32<	R	32<	R	2	S
G20AB22	2929	17	**S**	12	R	22	S	21	S	15	R	32	R	32<	R	2	S
G20AB08	3326	6	R	6	R	6	R	25	**S**	18	S	32	R	32	R	2	S

DISK: disk diffusion method; MIC: minimum inhibitory concentration using the microplate dilution method; (S): susceptible, (I): intermediate, (R): resistant

[Breakpoint]

Amikacin: ≥17 mm (S), 15-16 mm (I), ≤14 mm (R)

Gentamicin, Tobramycin : ≥15 mm (S), 13-14 mm (I), ≤12 mm (R)

Minocycline: ≥16 mm (S), 13-15 mm (I), ≤12 mm (R)

Tigecycline: ≥18 mm (S), <18 mm (R), the EUCAST criteria for *E. coli*

Imipenem, Meropenem: ≤2 μg/mL (S), 4 μg/mL (I), ≥8 μg/mL (R)

Colistin : ≤2 μg/mL (S), >2 μg/mL (R)

* : EUCAST V13.0 Breakpoint

** : Resistance genes are present, but there is susceptibility, which is shown in **bold**.
